# Carvacrol Essential Oil as a Neuroprotective Agent: A Review of the Study Designs and Recent Advances

**DOI:** 10.3390/molecules30010104

**Published:** 2024-12-30

**Authors:** Fahad Khan Tareen, Laura Catenacci, Sara Perteghella, Milena Sorrenti, Maria Cristina Bonferoni

**Affiliations:** Department of Drug Sciences, University of Pavia, Viale Taramelli 12, 27100 Pavia, Italy; fahadkhan.tareen01@universitadipavia.it (F.K.T.); laura.catenacci@unipv.it (L.C.); sara.perteghella@unipv.it (S.P.); mariacristina.bonferoni@unipv.it (M.C.B.)

**Keywords:** carvacrol, neuroprotection, Parkinson, multiple sclerosis, Alzheimer, traumatic brain injury, epilepsy, migraine

## Abstract

Neurodegenerative diseases were mostly perceived as diseases of ageing populations, but now-a-days, these diseases pose a threat to populations of all age groups despite significant improvements in quality of life. Almost all essential oils (EOs) have been reported to have some neuroprotective abilities and have been used as supplements for good mental health over the centuries. This review highlights the therapeutic potential of one such monoterpene phenolic EO, carvacrol (CV), that has the potential to be used as a main therapeutic intervention for neurodegenerative disorders. Three libraries, Google Scholar, PubMed, and ScienceDirect, were explored for research studies related to the neuroprotective roles of CV. All the research articles from these libraries were sorted out, with the first article tracing back to 2009, and the latest article was published in 2024. The positive effects of CV in the treatment of Alzheimer’s and Parkinson’s Diseases, multiple sclerosis, ischemia, and behavioural disorders have been supported with evidence. This review not only focused on study designs and the pharmacological pathways taken by CV for neuroprotection but also focused on demographics, illustrating the trend of CV research studies in certain countries and the preferences for the use of in vitro or in vivo models in studies. Our review provides useful evidence about the neuroprotective potential of CV; however, a lack of studies was observed regarding CV encapsulation in proper dosage forms, in particular nanoparticles, which could be further explored for CV delivery to the central nervous system.

## 1. Introduction

**Figure 1:**
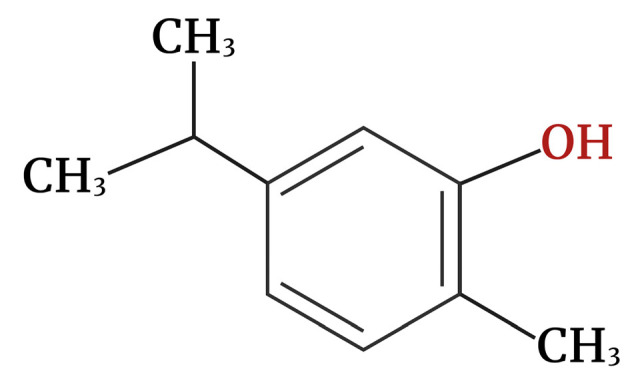
Chemical structure of carvacrol.

Carvacrol (CV), or cymophenol (2-methyl-5-propan-2-ylphenol, Figure 1), is a monoterpene phenolic compound obtained from the essential oils (EOs) of members of the Labiatae family, including Origanum, Satureja, Thymbra, Thymus, and Corydothymus [1]. Its boiling point is 237–238 °C, and it melts at 1 °C. The density of CV ranges from 0.976 g/cm^3^ at 20 °C to 0.97  g/cm^3^ at 25 °C. It is not soluble in water but is highly soluble in ethanol, carbon tetrachloride, and diethyl ether. The biological activities of CV have been shown in different in vivo and in vitro studies including antioxidant, antiseptic, anticarcinogenic, anti-inflammatory, antidiabetic, immunomodulatory, antimicrobial activity, antispasmodic, antibacterial, and growth promoter activities [2].

EOs have long been utilised in folk medicine. Known as ethereal or volatile oils, EOs are aromatic oily liquids derived from plant parts and utilised to flavour food. EOs are “essential” because they include the scent and botanical qualities. Antibacterial, antioxidant, antiviral, insecticidal, and other biological properties were found in these volatile oils. Some of these oils are utilised for cancer treatment, food preservation, aromatherapy, and perfumery. EOs’ antibacterial and antioxidant activities underpin many foods preservation and natural, pharmaceutical, and alternative medicine applications. An alternate wound healing method is aromatherapy, which uses EOs’ aromatic components [3]. Currently, there are around 3000 EOs that have been identified, with 300 of them being of commercial significance. These oils are particularly vital for industries such as pharmaceuticals, agriculture, food, hygiene, cosmetics, and perfumes. Certain EOs have been observed to possess specific therapeutic characteristics that are believed to prevent or potentially treat certain organ dysfunctions or systemic illnesses [4]. EOs are complex natural combinations of secondary plant metabolites with low-molecular-weight chemical components at varying quantities; terpenes, terpenoids, aromatic, and aliphatic derivatives predominate. Despite their historical benefits, plant derivatives are still gaining attention for their medicinal potential due to their natural source and vast range of pharmaceutical applications [5]. Many prosperous enterprises are currently engaged in the development of medications, nutraceutical products, and intermediate supplements using EOs as a key component. EOs are manufactured at a yearly rate of over 70,000 metric tonnes in several countries including the USA, Brazil, India, China, Bangladesh, Indonesia, Nepal, Thailand, Sri Lanka, South Africa, Egypt, Malaysia, France, Spain, Italy, Australia, Germany, and Russia. There are over three hundred EOs, including ajowan, anise, basil, camphor, celery sage, chamomile, clove, citronella, coriander, corn mint, cumin oils, dill, eucalyptus, fennel, lavender, lemon, orange oil, peppermint, thyme, tarragon, and others, that are classified based on their commercial and therapeutic worth. Certain specific EOs and their constituents are utilised as antiseptics, food preservatives, and dental root canal sealers due to their inherent antibacterial qualities. Additionally, several EOs are employed in agriculture for purposes such as biofertilisation, crop protection, natural pest control, germicidal activity, weed eradication, and more. EOs’ market worth is predominantly derived from its medicinal potential, as well as criteria such as quality and purity, resulting in an exceptionally high market value [6]. EOs have been the subject of study for almost 60 years, but in recent decades, there has been a surge of interest in them due to a desire to find natural therapies. For thousands of years, EOs have been recognised and utilised for their therapeutic powers in both medicinal and ritual practices, even dating back to prehistoric times [7]. EOs are increasingly used for recuperation and other beneficial effects. The global EOs’ market is expected to rise to USD 3226.2 million by 2025. The positive impact of aromatherapy treatments, combined with the trend of Generation X’s and Millennials’ interest in body health and awareness of natural medicine, drives high demand among therapists and spas, especially for EOs, which have a 70% market share [8].

Neurological diseases are characterised by the impairment and decline of neuronal cells, resulting in functional and sensory deficits. Multiple variables, including environmental influences, genetic predisposition, and oxidative toxicity, contribute to the development of these disorders. Oxidative stress plays a significant role in the development of dementia. The accumulation of reactive oxygen species (ROS) causes harm to biomolecules such as DNA, lipids, and proteins, leading to cellular dysfunction if left unaddressed with implications for neurological problems [9]. Furthermore, the United Nations reported that 1 in 11 people were over 65 in 2019, and by 2050, the number will virtually treble to 1 in 6. Neurodegenerative diseases like Alzheimer’s disease (AD) and Parkinson’s disease (PD) are rising as the global population ages. Note that dementia cases in developed countries are expected to climb from 13.5 million in 2000 to 21.2 million in 2025 and 36.7 million in 2050. AD now kills as many people as stroke, the third biggest cause of death worldwide. Unfortunately, we still do not have a complete understanding of AD and other neurodegenerative disorders’ pathogenesis, early diagnosis signs, or viable treatments. The worst part is that AD patients are frequently diagnosed 10–20 years after symptoms occur, making it nearly impossible to prevent or delay disease development. We also face comparable issues with PD, the second most common neurodegenerative illness after AD [10]. There is a need and a lot of opportunities to employ EOs in nanomedicine and to unlock their true potential in healing and curing central nervous system (CNS) diseases. The trend in exploring EOs is emerging among researchers in neurological disorders because of their unwavering positive outcomes [9]. This review article is focused on exploring the documented benefits of CV in neurological disorders.

## 2. Methodology

Only research articles in English related to disorders of the brain and spinal cord were considered for this review by using the keywords ‘brain’, ‘autoimmune’, ‘neuron’, ‘myelin’, ‘spinal’, ‘neuroprotection’, ‘CNS’, ‘multiple sclerosis’, ‘Alzheimer’, ‘Parkinson’, ‘anxiolytic’, and ‘antidepressant’ in relation to ‘Carvacrol’ and ‘5-Isopropyl-2-methylphenol’. The libraries explored were Google Scholar, PubMed, and ScienceDirect. All the articles were considered depending on the research relativity according to the main theme of this review. The first study related to CV activity analysis in brain or CNS disorders was published in 2009, and a total of 59 articles were available to date from the above-mentioned libraries which were selected for review.

## 3. Results

Among the 59 articles, 7 studies were in-vitro based, 49 studies were based on in vivo animal models, and 3 were a combination of both in vitro and in vivo methods. For PD, a total of 6 articles were found; five involved only in vivo studies, whereas one had a combination of both in vitro and in vivo studies (Table 1). Moreover, six studies were selected for the treatment of AD, among which two were in vitro based, three were in vivo based, and one was a combination of both (Table 2). For multiple sclerosis (MS), only three studies were recorded for CV activity and were related to in vivo models (Table 3). Seven articles were related to traumatic brain injury (TBI) and spinal cord injury (SCI); all were in vivo based except one which was based on an in vitro cell culture model (Table 4). Eight articles for epilepsy, migraine, and cerebrospinal ischemia were based on in vivo studies (Table 5). Table 6 represents 10 articles (2 in vitro; 8 in vivo) demonstrating the neuroprotective effect of CV against certain drugs and chemical toxins. Six in vivo studies were related to CV’s neuroprotective effects on anxiety, depression, and behavioural/cognitive problems and are summarised in Table 7. Four articles related to the attenuative effects of CV in LPS-challenged animal models are summarised in Table 8. Another nine related neuroprotective studies of CV are compiled in Table 9.

## 4. Discussion

Most of the publications in this review were found to be from Iran, China, and Brazil. This could be attributed to two aspects: a strong belief in traditional natural treatment remedies and the availability of source plants in these countries. Iran boasts a wealth of cultural heritage, encompassing a sophisticated traditional medical system that has deep historical roots dating back to the Assyrian and Babylonian civilisations. Contemporary ethnomedical practices are the result of the accumulated wisdom of indigenous communities who have passed down their knowledge of cures for various diseases through countless generations over thousands of years. Traditional medicine knowledge serves as a significant source of inspiration in the creation of new medications and therapeutic procedures [70]. The southern region of Iran is home to the endemic plants *Satureja khuzistanica* and *Satureja rechingeri*. These species are CV-rich and biologically active. This subshrub has a branching stem about 30 cm high, is densely leafy, and is widely ovaiate-orbicular with white hairs. It is utilised as a traditional medicine for its analgesic and antibacterial effects. *S. khuzestanica* EO (SKEO) contains CV, antioxidant, and anti-thyroid flavonoids [71,72]. On the other hand, *Lippia origanoides*, commonly referred to as “salva-do-Marajó” in the northern part of Brazil, is a fragrant plant utilised by local inhabitants as a culinary spice, serving as a substitute for oregano. Wild specimens of L. origanoides found in the Lower Amazon River region of Brazil have yielded EOs that are rich in CV and possess antibacterial properties against clinically significant human diseases [73]. Moreover, “Shennong’s Herbal” is an ancient medical book that originated from the Chinese tradition and dates to 2700 B.C. It provides detailed instructions on how to use 365 different herbs. China remains the foremost global producer of EOs [74]. In traditional Chinese medicine, herbs rich in CV have been used for topical treatments. CV is present in the EOs of various plants native to China, such as *Mosla chinensis* Maxim, *Thymus vulgaris* L, *Piper nigrum* L, and *Mentha haplocalyx* Briq [75,76].

Among the selected articles, it can be observed that there are two routes of administration prominently used for CV: the oral route and the intraperitoneal route. The oral route was often termed as ‘oral gavage’ and considered a standard method to deliver the test formulation directly into the stomach of rodents. On the other hand, the intraperitoneal (IP) injection route was used more than any other route of administration owing to its easy application with no requirement for highly trained or specialised personnel to perform it. The route of administration has a crucial role in determining the final pharmacokinetics, pharmacodynamics, and toxicity of pharmacological drugs. The primary methods of drug delivery in laboratory animals are the intravenous (IV), subcutaneous (SC), IP, and oral routes. Each route has its own advantages and disadvantages, which vary based on the specific goals of the investigation. The IP route, often employed in rat investigations, involves the injection of a pharmacological substance into the peritoneal cavity. This technique is easily mastered and efficiently minimises stress for animals. The procedure entails positioning the mouse on its back, with its head lower than the rest of its body and inserting a needle into the lower section of the abdomen at an approximate angle of 10 degrees. Care must be taken to prevent unintentional puncture of the internal organs. This approach allows for the safe administration of significant quantities of solution (up to 10 mL/kg) to rodents, which can be beneficial for substances that have low solubility. This technique is particularly prevalent in chronic investigations that involve mice, where repeated IV access is difficult. Typically, IP administration is favoured over the oral route for biological medicines to prevent exposure to the gastrointestinal tract and probable degradation or alteration of biopharmaceuticals [77]. One often-used method for administering substances to mice in experiments is oral gavage, which entails inserting a feeding needle via the mouth and into the oesophagus. Oral gavage is the most direct method to accurately administer substances into the gastrointestinal tract of mice [78]. Oral gavage is the most used approach for precise oral dosing in rodent experiments. With a qualified operator, the process is fast and delivers a precise amount of a drug directly into the stomach for absorption. Gavage is useful when the substance cannot be fed or is unpleasant [79].

Overviewing the selected articles, it can be seen that CV was mostly emulsified with tween-80 and tween-20 as the surfactant and dissolved in a vehicle (saline, distilled water, and DMSO) and delivered to test subjects as a simple homogenous solution form. In two of the articles, peanut oil [64] and olive oil [52] were used as vehicles for CV. Test subjects were either rodents (mice, rats, or rabbits) or cell cultures. Only two studies encapsulated the CV in a specialised dosage form, i.e. ‘nanoemulsion’, and analysed the activity in different in vitro and in vivo models [19,61]. From the selected articles, it was observed that most of the studies employed the conventional in vivo analysis to evaluate or compare the effect of CV. Only a few studies utilised the cell cultures in vitro analysis for their studies. This trend can be attributed to numerous compelling reasons. Animals are more complete models to evaluate the effects of substances on CNS disorders. Among the most used animal models, mice share 80% of their genetic material in common with humans. Rodents, being highly analogous to people, are susceptible to diseases that bear resemblance to those affecting humans [80].

### 4.1. Neuroprotective Ability of CV

**Figure 2:**
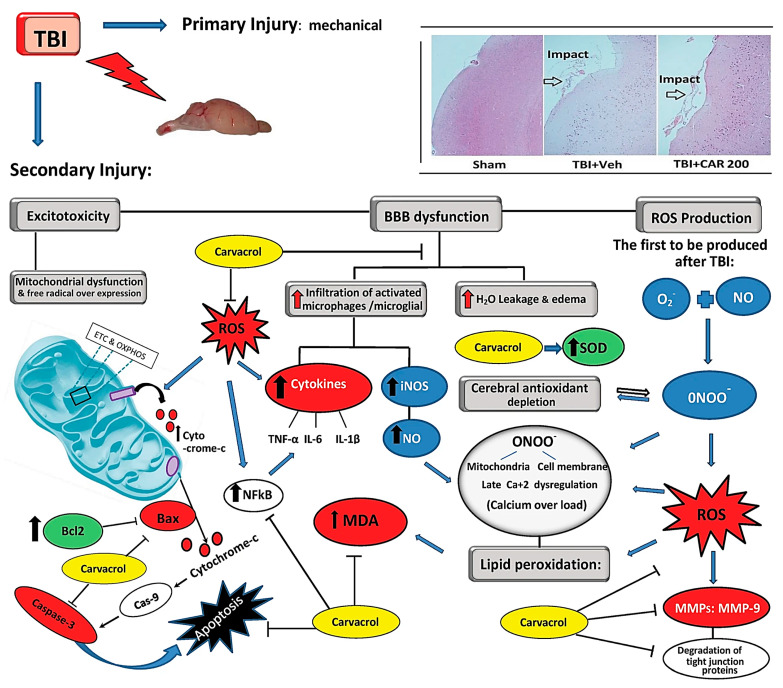
Illustration of the role of CV in attenuating the damage caused by traumatic brain injury in a mouse model. TBI induces secondary damage through excitotoxicity, BBB disruption, mitochondrial dysfunction, and excessive free radical production. Elevated intracellular Ca²⁺ levels from mitochondrial dysfunction lead to increased NO and ROS, causing oxidative stress and impairing antioxidants. BBB permeability also causes vasogenic oedema and infiltration of activated microglia, producing NO and peroxynitrite, which contribute to lipid peroxidation, DNA damage, and protein oxidation. Carvacrol may mitigate these damaging processes by inhibiting ROS. Abbasloo et al. demonstrated that CV has an influence on reverting the oxidation, inflammatory, and apoptotic pathways in a TBI model, simultaneously [30].

CV has been appraised in the literature for its antioxidant, anti-inflammatory, and anti-apoptotic activity (Figure 2), not only in neurodegenerative diseases but also in other chronic pathological conditions like cancer.

#### 4.1.1. Antioxidant Activity of CV

A loss of equilibrium between the production and accumulation of ROS and reactive nitrogen species (RON) in neuronal cells followed by failure of cellular mechanisms to eliminate them is referred to as ‘oxidative stress’ [81]. Under normal circumstances, a ROS/RON imbalance triggers a cellular antioxidant defence system through enzymatic and non-enzymatic pathways to scavenge the free radicals [82]. Among the enzymatic defence pathways against oxidative damage, superoxide dismutase (SOD), catalase (CAT), glutathione peroxidase (GPx), NADPH-quinone oxidoreductase-1 (NQO1), heme-oxygenase (HO-1), thioredoxin (Trx), and sulfiredoxins (Srx) scavenge and re-balance the cells’ internal homeostasis. On the other hand, vitamin C, vitamin E, β-carotene, uric acid, and a tripeptide glutathione (GSH) augmented with a thiol are notable antioxidant components of cells [83,84]. ROS is a collective term with hydrogen peroxide (H_2_O_2_) and the superoxide anion radical (O2·−) as the main redox signalling agents [85]. CV has been regarded as a potent antioxidant EO attributed to the presence of a hydroxyl group (-OH) as well as methyl and isopropyl groups. CV presents a system of delocalised electrons due to these functional group substituents, making it an effective sensor for free radicals. Being a weak acid, CV donates hydrogen atoms to free electron pairs, neutralising a free radical species [86]. On the other hand, as an active redox agent, CV has been reported to donate electrons to scavenge free radicals as well [87]. These redox scavenging properties make it an efficient active substance that can be utilised as a food supplement as well as in pharmaceuticals.

#### 4.1.2. Anti-Inflammatory Activity of CV

**Figure 3:**
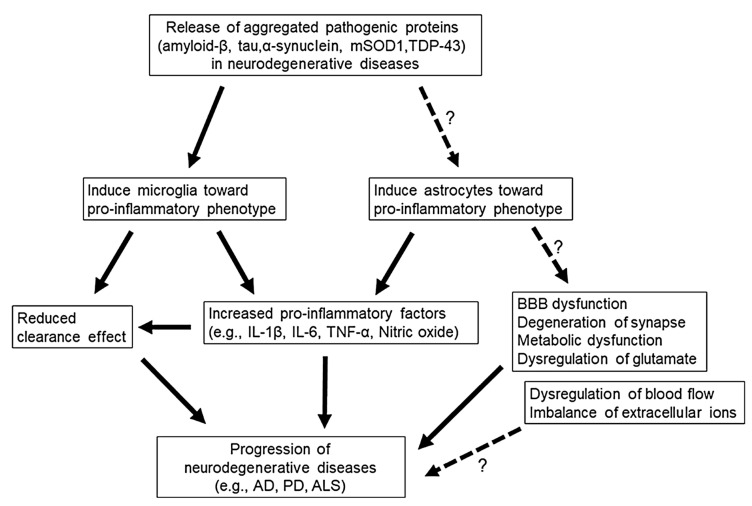
Kwon and Koh illustrated the pathways in the progression of neurodegenerative diseases by the release of pathogenic proteins in the brain, focusing on the role of microglia and astrocytes. The release of aggregated pathogenic proteins like amyloid-β, tau, α-synuclein, mSOD1, and TDP-43 triggers microglia and astrocytes to adopt pro-inflammatory phenotypes. This promotes the release of pro-inflammatory factors, impairing synaptic function, blood–brain barrier integrity, and metabolic processes, driving neurodegenerative disease progression. A dotted line with a question mark indicates a potential relationship, where direct evidence for the association is lacking [90].

Neuroinflammation is a protective defence system of the brain against any insults or damage; however, it could turn into neurodegeneration in the case of chronic inflammatory conditions. The key regulators of the neuronal immune system are microglia and astrocytes (Figure 3). A balance between two distinctive phenotypes of these two glial cells determines the fate of neurons towards protection or destruction. The activation of the M1 phenotype of microglia and the A1 phenotype of astrocytes is associated with neurotoxicity, and M2 and A2 are related to neuroprotection [88,89].

CV has been reported to suppress the expression of prostaglandins, especially PGE2, via the arachidonic acid pathway, with inhibition of cyclogeneses, COX1, and COX2, initiating robust anti-inflammatory activity [90]. Moreover, CV has a documented attenuation activity for LPS-induced inflammation by inhibiting ERIK-1/2 phosphorylation [91]. Moreover, CV was found to be associated with inhibiting the translocation of NF-kß (p65) from the nucleus to the cytoplasm but had no effect on p38. Among the inflammatory cytokines, matrix metalloprotease (MMP-1, MMP-3, and MMP-13) production was also hindered by CV. On the other hand, the production of the neuroprotective cytokines IL-10 and TGFß was supported by CV, thus augmenting the neuronal anti-inflammatory innate defence system [92].

#### 4.1.3. Anti-Apoptotic Activity of CV

**Figure 4:**
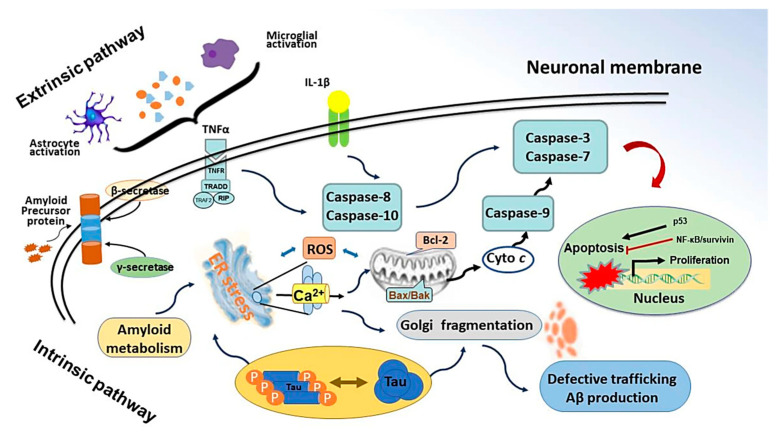
Graphical representation of neuronal apoptosis by intrinsic factors and extrinsic factors. The intrinsic apoptotic pathway in neurons is triggered by stress signals, leading to mitochondrial cytochrome c release, caspase activation, and cell death. Moreover, the extrinsic apoptotic pathway in neurons is activated by death receptor signalling, leading to the activation of caspase-8 and downstream caspase cascades. This pathway contributes to neuronal cell death in response to external stimuli [96].

Oxidative stress leading to inflammation often ends with apoptosis. Neuronal apoptosis is followed by intrinsic or extrinsic factors as shown in Figure 4. Intrinsic signalling of programmed cell death starts with the upregulated expression of BH3-only proteins. These proteins downregulate BcL2 expression, an anti-apoptotic protein [93], and upregulate the expression of Bax proteins. This leads to leaching of cytochrome c, which activates APAF-1 which starts activating the caspases via procaspase 9. On the other hand, extrinsic factors start by activating caspase 8. Both these pathways lead to caspase 3, also known as the ‘death executioner’ protein that results in the end of the cell [94,95]. CV plays an important role in neuronal apoptosis. CV has been reported to downregulate Bax and caspase 3 proteins and to upregulate BcL2 proteins, attenuating apoptosis in neuronal cells [31,44,50]. Moreover, CV has been reported in the limited literature as a neurotrophic substance that has the potential to initiate neurite outgrowth independent of nerve growth factor (NGF) [62].

### 4.2. Need of Suitable Dosage Forms for CV

**Figure 5:**
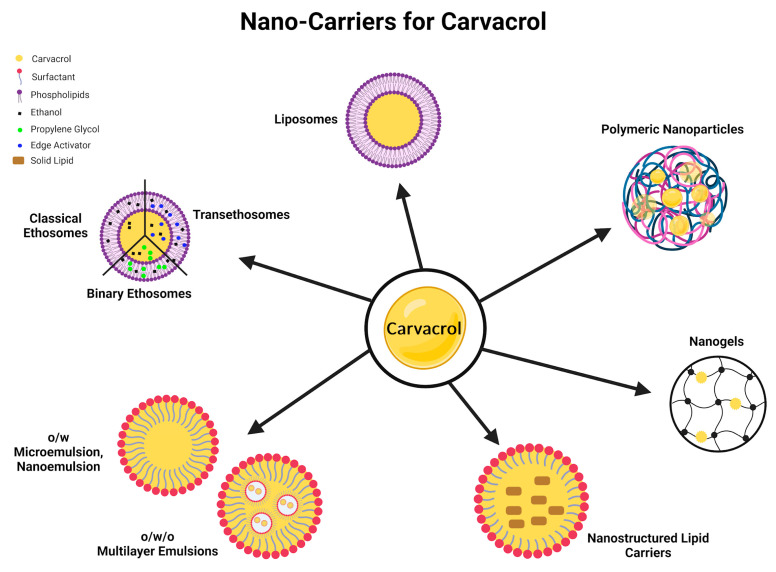
An illustration of nano-carriers for carvacrol.

Only two studies in our literature survey optimised CV in a dosage form (nanoemulsions) and compared their neuroprotective efficacy against a conventional solution form or directly as an oil administered to mice models. In both articles, the authors reported that CV in a nanoemulsion had a notably increased efficacy and better stability as compared to a CV solution [19,61]. EOs are active ingredients, each having a diverse therapeutic profile, but their activity is limited due to low environmental stability, low solubility, unpredictable pharmacodynamics, high toxicity at higher doses, and low patient adherence due to their taste or odour as a pure oil [97,98,99]. Encapsulating an EO protects it from harsh environment, saves it from volatility, and provides a controlled/sustained/targeted release of the EO for an efficient therapeutic efficacy. Apart from the taste masking of EOs in a proper dosage form via either a micro or nano vehicle, EOs can be administered at high doses with minimised toxic effects [100,101]. Souza et al. [102] compiled a literature review about the available nano dosage forms encapsulating CV for antibacterial, antifungal, anti-inflammatory, antitumor, and some biological activities. He emphasised that nanotechnology can be used as a tool to enhance the therapeutic potential of CV, and the limitations associated with EOs can be eliminated by using an appropriate nano vehicle, as illustrated in Figure 5 [102]. Most of the essential oils face challenges in crossing the BBB due to their large molecular weight [103]. However, CV is a small molecule weighing only 150 Da, so permeability is apparently of no concern. The focus for CV formulation is to control the release of CV across the BBB to avoid any toxicity. Furthermore, there is still room for investigation regarding the interactions between CV and different moieties in the blood, epithelium, and body fluids that could hinder the efficacy of CV’s therapeutic action at the target site (brain). A nano formulation can be the answer to these challenges as it provides a safe passage for CV across the systemic circulation to the BBB where it can cross and exerts its neuroprotective action.

Moreover, to date, there is a research gap in exploring the true neuroprotective potential of CV encapsulated in a nano/micro dosage form. We are positive that giving a proper vehicle to this EO can turn the tables when it comes to neuroprotection against notorious brain diseases.

## 5. Conclusions

In conclusion, this review highlighted the therapeutic potential of CV EO in neurodegenerative disorders. CV does not rely on a single mechanism to initiate neuroprotection; rather, it works simultaneously on multiple pathways working as an antioxidant, anti-inflammatory, and anti-apoptotic agent. Moreover, a few studies reported on the gene modulation ability of CV as well. However, more systematic studies are needed to fully understand the therapeutic potential of CV encapsulated in a pharmaceutical dosage form. Dosage of CV was also deemed a contradictory point among the reviewed studies as some studies reported a higher dose as beneficial and some reported a lower dose as more effective. In summary, it is evident from the current review study that CV can provide neuroprotection, and future studies should focus on integrating pharmaceutical nanotechnology in CV formulation designs. In particular, intranasal drug delivery is a widely explored administration route for the delivery to the CNS and could be explored to evaluate the neuroprotective effect of CV at low doses to avoid toxicity and to achieve faster effect.

## Figures and Tables

**Table 1:** Summary of study designs and effect of CV on Parkinson’s disease.

No.	Study Type	Ref.	Formulation Type	Doses of CV	Experiment Timespan (Days)	Test Subject	Route	Analyses Techniques	Key Findings and Remarks
1	In vivo	[11]	Solution (saline)	40 mg/kg	15	Male mice (C57BL/6)	IP	Immunohistochemistry Western blots Behavioural analysis (cylinder test)	Promising neuroprotective role of CV was illustrated in the 6- hydroxy-dopamine model (6-OHDA) of PD. Reduction in Caspase-3 to control levels. Non-specific blocking effect on transient receptor potential melastatin 7 (TRPM7) channels.
2		[12]	Solution	12.5 and 25 mg/kg	28	Male Wistar Rats	IP	Behavioural tests (catalepsy test, open field test, vacuous chewing movements) Tyrosine hydroxylase (TH) immunohistochemistry Image analysis	CV exerted a protective effect in PD model. Cataleptic behaviour and vacuous chewing movements were prevented. However, decreased open-field locomotor activity did not show any improvement by CV.
3		[13]	Solution	25 mg/kg	49	Male Wistar Rats	IP	Apomorphine-induced rotation test Behavioural test (passive avoidance memory by a shuttle box) Lipid peroxidation levels Total thiol concentration	CV in conjunction with treadmill exercise helped in treating the neurobehavioral deficits associated with PD. Furthermore, CV therapy and exercise decreased rotating behaviour and enhanced memory deficits. Lowered lipid peroxidation levels and elevated total thiol concentration in the striatum and/or hippocampus.
4		[14]	Solution	10 mg/kg	14	Male Spraque-Dawley rats	IP	Immunohistochemical analysis Gene expression analyses Western blot analysis	CV might be associated with the protection of dopaminergic neurons via the reduction in reactive astrogliosis. Modulation in expression of TRP channels was observed. Expression of TRPA1 was upregulated with CV treatment in PD model. However, expression of TRP7 was unaffected by CV.
5		[15]	Solution	25 mg/kg, 50 mg/kg, 100 mg/kg	21	Swiss Albino Mice	PO	Behavioural parameters (open-field test, rotarod test) Immunofluorescence Western blotting	CV was reported to be associated with increased antioxidant activity and a neuroprotective effect in a PD model. A reduction in inflammatory cytokines, neurotoxicity, oxidative stress, and motor impairment caused by Rotenone was observed in mice treated with CV.
6	In-vitro and in vivo	[16]	Solution	10, 15, and 20 mg/kg	15	PC12 cell-based neuronal model and Male Albino Wistar Rats	IP	Cell viability assay ROS Assay Lipid peroxidation assay Annexin-V assay Behavioural tests (apomorphine-induced rotation, pole test, catalepsy test, beam walking, rotarod test, open-field test) Biochemical analysis	CV exerted antioxidant activity by inhibiting ROS production in dopaminergic cells, a potential neuroprotective agent for PD. Authors suggested that this activity might be related to the ability of CV to scavenge ROS or trigger the intracellular antioxidant defence system. Moreover, CV improved locomotor behaviour.

PO = Per os (Oral Gavage), IP = Intraperitoneal.

**Table 2:** Summary of study designs and effect of CV on Alzheimer’s disease.

No.	Study Type	Ref.	Formulation Type	Doses of CV	Experiment Timespan (Days)	Test Subject	Route	Analyses Techniques	Key Findings and Remarks
1	In vitro	[17]	Solution	1 to 1000 µM	2	PC12 cell-based neuronal model	-	MTT cell viability assay Measurement of ROS generation Kinase activity assay	CV along with thymol demonstrated a protective effect in PC12 cells against Aβ_25–35_. Moreover, increased antioxidant activity and expression of protein kinases C (PKC) were proposed to be related to the protection of memory and cognitive functioning.
2		[18]	Solution	5, 10, 25, 50, and 100 µg/mL	1	Human neuroblastoma (SH-SY5Y) cancer cells	-	Analysis of cholinesterase and a-amylase inhibition Enzyme activity Hydrogen peroxide scavenging assay MTT assay	*p*-cymene and CV exhibited anti-enzymatic properties and may function as neuroprotective agents against oxidative stress in AD patients. The inhibitory impact of CV on acetylcholinesterase (AChE) via the reduction in caspase-3 expression was found to be fourfold greater than that of p-cymene. The authors reported that the activity of CV was due to presence of an OH group in its structure.
3	In vivo	[19]	Oil and nanoemulsion	20 µL/kg of either oil or nanoemulsion	30	Male Wistar Albino Rats	PO	Analysis of brain cholinesterase (quantitative colorimetric kinetic assay) Analysis of brain monoamines Determination of urinary 8-hydroxy-2′-deoxyguanosine (8-OHdG), immunohistochemistry of brain cyclooxygenase	CV oil and CV nanoemulsion were found to be significant in their ability to reverse AlCl_3_-induced brain AD, which could be attributed to the antioxidant and anti-inflammatory properties of CV, modifying the effects of oxidative stress. In addition, it was noted that CV nanoemulsions provided a more effective and efficient method of delivering CV across the blood–brain barrier and ameliorating any brain alterations as compared to oil.
4		[20]	Solution	0.5, 1, or 2 mL/kg	5	Male Wistar Albino rats	IP	Behavioural test (Morris water maze test) Kinase activity assay Western blot Histopathological examination	In an AD model of rat brains, thymol and CV enhanced learning and memory deficits by stimulating hippocampal PKC signalling. As the modulation of PKC activity has the potential to improve cognitive function and potentially alter the pathophysiology of AD, this upregulation of PKC by CV and thymol could prove to be potential therapeutic strategies in AD.
5		[21]	Solution	50 mg/kg	56	Male Wistar rats	PO	Determination of population spike (PS) amplitude and field excitatory postsynaptic potentials slope	CV or p-cymene alone was found to be effective in preventing synaptic plasticity impairment in an AD model. A potential interaction is reported between CV and p-cymene, as their combined therapy did not prevent the adverse outcomes of Aβ_1–42_ on synaptic plasticity.
6	In vitro and in vivo	[22]	Solution	100, 200, and 300 µM for cell lines and 1 mg/kg IP injection for rats twice daily	6	SH-SY5Y neuroblastoma cells and Male Wistar Rats	IP	MTT assay Determination of oxidative stress-related biomarkers and Tau peptide in cell culture supernatant Assay procedures for SOD, MDA, and Tau peptide Assay procedure for H_2_O_2_	CV prevented the release of LDH. CV controlled the levels of MDH and H_2_O_2_ in vitro; however, it had no effect on these parameters in vivo. CV-treated rats demonstrated memory impairment in vivo. In a nutshell, CV is a multitarget pharmacological agent that shows potential in treating AD by inhibiting AChE activity, neuronal toxicity, oxidative stress, neuroinflammation, and memory problems linked to the disease’s aetiology.

PO = Per os (Oral Gavage), IP = Intraperitoneal.

**Table 3:** Summary of study designs and effect of CV on multiple sclerosis.

No.	Study Type	Ref.	Formulation Type	Doses of CV	Experiment Timespan (Days)	Test Subject	Route	Analyses Techniques	Key Findings and Remarks
1	In vivo	[23]	Solution	5 and 10 mg/kg	21	Female C57BL/6 mice	IP	Histopathological analysis Proliferation assay Cytokine assay	Lower doses (5 mg) of CV were found to be more effective than higher doses (10 mg) in mitigation of autoimmune encephalomyelitis (EAE) in MS model. In particular, the infiltration of leukocytes into the CNS was reduced by CV, and the production of pro- and anti-inflammatory mediators were modulated in the mouse model.
2		[24]	Solution	20 mg/kg	28	Female Lewis Rats	IP	Histological assessment Immunohistofluorescence RNA extraction and quantitative RT-PCR	The remyelination rate increased in the CV-treated group. CV increased the expression of OLIG2 which is an important transcription factor responsible for remyelination in degenerated lesions.
3		[25]	Solution	25 mg/kg	29	Female Lewis Rats	IP	Histopathological analysis Fluorescence immunohistochemistry RNA extraction and RT-PCR	CV was reported to be associated with increasing the gene expression for myelin regeneration and decreasing the gene expression responsible for inflammatory cytokines in CNS. CV improved the pathophysiological symptoms related to EAE in MS mice model.

IP = Intraperitoneal.

**Table 4:** Summary of study designs and effect of CV on traumatic brain injury and spinal cord injury.

No.	Study Type	Ref.	Formulation Type	Doses of CV	Experiment Timespan (Days)	Test Subject	Route	Analyses Techniques	Key Findings and Remarks
1	In vitro	[26]	Solution	0.5 and 1 mM	<1	Cortical neurons cell culture	-	Lactate dehydrogenase (LDH) assay Calcium imaging Real-time RT-PCR Measurement of caspase-3 activity Western blot analysis	CV treatment decreased intracellular Ca^2+^ concentration following traumatic neuronal injury, enhancing neuronal viability and reducing apoptosis. The authors proposed that the effects might be linked to a strong positive feedback loop of intracellular Ca^2+^ regulation and decreased activation of the nNOS pathway by CV.
2	In vivo	[27]	Solution	75, 750 mg/kg, and 3.75 g/kg	>21	Sabra Mice and C57BL/6 mice (wild type)	IP	Neurobehavioral tasks (NSS score)	CV demonstrated neurological recovery in TBI models when used synergistically with TRPC1 elimination. CV significantly improved functional recovery of mice at the lowest dose and had no effect at higher doses.
3		[28]	Solution	50 mg/kg	7	Male Sprague Dawley rats	IP	Free Zinc analysis Evaluation of hippocampal degenerating neurons Immunohistochemistry Immunofluorescence analysis Modified neurological severity score	The current investigation indicated that CV administration reduced the overexpression of TRPM7 and the build-up of free zinc in hippocampal neurons after TBI. The decrease in free zinc accumulation resulted in a reduction in degenerating neurons, dendritic injury, oxidative stress, and glutathione depletion after TBI. Moreover, it was observed that CV therapy not only reduced microglial activation and postponed neuronal death but also improved neurological outcomes after TBI.
4		[29]	Solution	100 and 200 mg/kg	1	Male Wistar Rats	IP	Brain water content (BWC) assessment Neurological outcome assessment Western blot analysis of apoptotic and inflammatory markers ELISA of inflammatory cytokines Immunohistochemistry	*Satureja khuzistanica* Jamzad EO (SKEO) have a total 4.5% of EO. CV makes up 94.16% of SKEO. CV therapy significantly inhibited the excessive production of pro-inflammatory cytokines in the brain, including IL-1β, TNF-α, and IL-6, and was reported to have more neuroprotective activity than SKEO.
5		[30]	Solution	100–200 mg/kg	1	Male Wister rats	IP	BWC assessment Determination of blood–brain barrier permeability Measurement of mean arterial pressure (MAP) Spectrophotometric assessment of MDA and SOD Total antioxidant capacity assessment Determination of ROS Western blot analysis of MMP-9, ZO-1, Occludin, and Claudin-5 expression	CV (200 mg) improved oxidative homeostasis and preserved the BBB which could promote behavioural recovery after TBI. The loss of ZO-1, occludin, and claudin-5 proteins after TBI was prevented by CV through an MMP-9 signalling pathway. The 200 mg/kg dose of CV did not induce any acute alterations in blood pressure, heart rate, or body mass, suggesting that it might be the most suitable dose for assessing the long-term effects of CAR on pathophysiological processes.
6		[31]	Solution	25, 50, and 100 mg/kg	46	Wistar Rats	IP	Evaluation of neuronal function recovery Assessment of water content in spinal cord tissues Measurement of MDA level and the activity of CAT, SOD, and GSH-Px. Immunoblotting Measurement of eNOS activity in spinal cords and plasma NO production Measurement of caspase-3 activity in spinal cord tissues	CV provided neuroprotection in spinal cord injury (SCI) rat models, dose dependently. The protective activity by CV was associated with a reduction in NO levels, Bax protein, and caspase 3. Moreover, levels of Bcl-2, an anti-apoptotic protein, were elevated by CV.
7		[32]	Solution	50 mg/kg	7	Male Sprague Dawley rats	IP	Behavioural tests (Basso-beattie-Bresnahan (BBB), inclined plane test, grid walk, and footprint) Measurement of BSCB disruption RNA isolation and RT-PCR Immunohistochemistry Western Blot Measurement of transendothelial electrical resistance Cell counting of viable ventral motor neuron Histological analysis of myelin and axon Measurement of lesion volume	CV was reported to inhibit the disruption of brain–spinal cord barrier (BSCB) after spinal cord injury. CV could have a direct neuroprotective effect by blocking TRPM7 channel. Anti-apoptotic and antioxidant activity of CV lead to improved functional recovery and inhibited the leakage of blood and inflammatory cytokines.

IP = Intraperitoneal.

**Table 5:** Summary of study designs and effect of CV on epilepsy, migraine, and cerebral ischemia.

No.	Study Type	Ref.	Formulation Type	Doses of CV	Experiment Timespan (Days)	Test Subject	Route	Analyses Techniques	Key Findings and Remarks
1	In vivo	[33]	Solution	75 mg/kg	10	Male Sprague–Dawley rats	IP	Seizure Analysis Immunostaining Rewarded alternating T-maze test	CV inhibited recurrent status epilepticus (SE) and early seizures in vivo, but it did not have a detectable effect on paired-pulse inhibition or the fibre salvo in the hippocampus, suggesting that it was not acting through sodium channel inhibition or GABA receptors. CV promoted marked neuroprotection possibly by its non-specific blocking effect upon TRPM7 channels.
2		[34]	Solution	100 mg/kg	<1	Male Wistar rats	IP	Biochemical evaluation of COX-1 and -2 in the hippocampal tissue	Lipopolysaccharide (LPS)-induced neuroinflammation, leading to seizures in epilepsy, was found to be countered effectively by CV treatment, which blocked the COX-2 pathways in hippocampus. However, seizures indexes by Pentylenetetrazol (PTZ) were found to have no effect from CV treatment.
3		[35]	Solution	25 and 50 mg/kg	14	Female Sprague–Dawley rats	-	In silico analysis Behavioural tests (thermal allodynia, mechanical allodynia, head-scratching, and light aversion) Antioxidant profile Imaging Immunohistochemistry Western Blot	The anti-migraine action of CV, illustrated specifically by behavioural tests, was reported to be mediated through anti-inflammatory and antioxidant pathways. CV exhibited binding affinities against various targets implicated in migraine pathology.
4		[36]	Solution	10, 20, and 40 mg/kg	<1	Male Sprague–Dawley rats	IP	Measurements of the levels of superoxide dismutase and malondialdehyde Western Blot qRT-PCR, enzyme-linked immunosorbent assay	CV has the potential to serve as a therapeutic agent for the treatment of cerebral ischemia injury owing to its antioxidant and anti-inflammatory properties. It was observed that CV inhibited the inflammatory response by inhibiting the NF-kB signalling pathway in rats with focal cerebral ischemia–reperfusion.
5		[37]	Solution	50 mg/kg	3	Male Sprague Dawley rats	IP	Evaluation of hippocampal degenerating neurons Zinc translocation Lipid peroxidation Microglial activation Transient receptor potential melastatin 7 channel regulation	CV provided neuroprotection by blocking TRPM7 pathways in rats with zinc-induced neurotoxicity. CV was hypothesised to reduce the translocation of intracellular zinc after global cerebral ischemia (GCI).
6		[38]	Solution	25, 50, and 100 mg/kg	14	Male gerbils	IP	Behavioural test (Morris water maze) NeuN immunohistochemistry Immunofluorescence MTT assay Oxidative stress measurements Iron measurements Western blot	CV protected the hippocampal neurons from degeneration by ischemia–reperfusion due to its antioxidant and anti-ferroptosis properties. This protective mechanism of CV was suggested to be partly the result of upregulation of glutathione peroxidase 4 (GPx4) activity which led to a reduction in lipid peroxidation.
7		[39]	Solution	100 mg/kg	2	Wistar rats	IV	Determination of motor deficit index Evaluation of total oxidative status Evaluation of total antioxidant status	CV could potentially protect motor functioning from neurological complications from spinal ischemia and reperfusion (SIR). However, total oxidative and antioxidative status levels after CV treatment in SIR was found to remain the same.
8		[40]	Solution	25 and 50 mg/kg	56	Male Wistar rats	PO	Behavioural tests (Morris water maze test) Histopathological examination Biochemical analysis (determination of MDA and DPPH activity in hippocampus tissue)	CV demonstrated neuroprotection of hippocampal ischemia in rats induced by chronic cerebral hypoperfusion (CCH). CV upregulated the activity of catalase and superoxide dismutase, resulting in reduced lipid peroxidation. Spatial learning and memory functioning were potentially recovered, probably via the antioxidant activity of CV.

PO = Per os (Oral Gavage), IP = Intraperitoneal, IV = Intravenous.

**Table 6:** Summary of study designs and neuroprotective effect of CV against drugs and toxic chemicals.

No.	Study Type	Ref.	Formulation Type	Doses of CV	Experiment Timespan (Days)	Test Subject	Route	Analyses Techniques	Key Findings and Remarks
1	In vitro	[41]	Solution	10 to 1000 µM	<1	Human neuroblastoma SH-SY5Y cell line	-	Cell viability and cytotoxicity assays Quantification of the production of O2−· and NO· Examination of the mitochondria-related apoptotic factors and cell death-associated parameters Quantification of enzyme activities Evaluation of the levels of ATP Measurement of the mitochondrial membrane potential (MMP) Examination of the levels of malondialdehyde (MDA) Protein carbonyl and 8-Oxo-2′-Deoxyguanosine (8-Oxo-dG) Determination of the levels of 3-Nitrotyrosine Measurement of the levels of Interleukin-1β (IL-1β) and Tumour Necrosis Factor-α (TNF-α) Quantification of the activity of Nuclear Factor-κB (NF-κB)	The deleterious effects of the pro-oxidant agent H_2_O_2_ were mitigated by CV pre-treatment through a mechanism that involved the enzyme HO-1. CV could assist in the preservation of the mitochondria, the body’s powerhouse, and thus, in turn, aids in the prevention of neurodegeneration, which could lead to brain diseases. However, authors suggested further in vivo studies to check the optimum therapeutic doses of CV since, at higher concentrations, CV was found to be intoxicating to cell lines.
2		[42]	Solution	10, 25, 50, and 100 mg/mL	2	Cortex neurons cell culture from Sprague Dawley rats; 24 h after birth	-	MTT assay Total antioxidant capacity (TAC) assay Total oxidant status (TOS) assay The cholinesterase activity assay Measurement of total thiol amount	Hydroxychloroquine use in the COVID-19 pandemic was found to be associated with neurodegenerative oxidative stress. 100 mg of CV was found to reduce the oxidative stress, induced by hydroxychloroquine, by 1.4 times. However, the anti-AChE activity of CV was proposed to be dependent on the concentration and the target tissues.
3	In vivo	[43]	Solution	73 mg/kg	2	Male Wistar albino rats	IP	Biochemical analysis	CV protected the sciatic nerve tissues against methotrexate (MTX)-induced oxidative stress better than pomegranate (POM) extract. However, both the CV and POM treatment groups demonstrated a decrease in pro-inflammatory responses in mice models.
4		[44]	Solution	25, 50, and 100 mg/kg	28	Male C57BL/6 mice	IP	Blood ethanol concentration Behavioural test (Morris water maze) Immunohistochemistry Hippocampal neuron viability assay and oxidative stress analysis Western blot Flow cytometric analysis Caspase-3 activity assay	CV protected hippocampal neurons owing to its antioxidant and anti-apoptotic properties. Ethanol-induced cognitive dysfunction was reversed by CV treatment especially at higher doses of 50 and 100 mg/kg in mice fed with a (35%) ethanol diet for 3 weeks prior to treatment. Moreover, CV protected against neuronal apoptosis possibly by upregulating Bcl-2 and downregulating Bax protein.
5		[45]	Solution	20 mg/kg	28	Male Wistar rats	PO	Total thiol content Malondialdehyde (MDA) concentrations Catalase (CAT) activity Superoxide dismutase (SOD) activity Brain index percentage	CV and *Zataria multiflora* (ZM) hydroalcoholic extract inhibited doxorubicin (DOX)-induced oxidative stress on the brain tissues of rats. Moreover, CV and ZM extract treatment for a month had a protective effect on overall systemic oxidative stress by DOX.
6		[46]	Solution	50 mg/kg	56	Male Albino rats	PO	Behavioural assessment (elevated plus maze test, forced swim test, Y-maze test) Biochemical Analyses (catalase enzyme activity, glutathione concentration, malondialdehyde concentration, acetylcholinesterase enzyme activity) Histopathology and immunohistochemistry	CV effectively countered the neurotoxic effects induced by propiconazole (PCZ) in rats. CV was able to ameliorate PCZ neurotoxicity; however, AChE activity was downregulated in both the control and treatment groups. CV tends to use phenolic hydroxyl groups of AChE to attach to other AChE enzymes, leading to the loss of enzyme function.
7		[47]	Solution	40 and 80 mg/kg	42	Male Wistar rats	PO	Biochemical assessment Oxidative stress and antioxidant status assessment Gene expression assessment by real-time polymerase chain reaction (RT-PCR) Gene expression Histopathological assessment	D-galactose-induced oxidative stress in the brain was reported to be inhibited by CV or eugenol (EU) treatment. A higher concentration of CV demonstrated a greater improvement in the hippocampus and lowered the necrosis levels more than the control and EU treatment groups. Overall, both CV and EU could be potentially used as anti-ageing substances due to their anti-inflammatory, antioxidant, and anti-apoptotic properties.
8		[48]	Solution	25 and 50 mg/kg	7	Male Sprague Dawley rats	PO	Assay of lipid peroxidation in brain tissue Assay of enzymatic and non-enzymatic antioxidants in brain tissue Assay of inflammation markers in brain tissue Assay of GFAP and MAO levels in brain tissue Assay of apoptotic markers in brain tissue Assay of oxidative DNA damage marker in brain tissue Real-time PCR analysis	CV could potentially ameliorate cadmium-induced neurodegeneration due to its antioxidant and anti-inflammatory properties. The neuroprotective role at both tested doses of CV showed no difference as such.
9		[49]	Solution	50 mg/kg	15	Male Wister rats	IP	Behavioural tests (gait score, thermal hyperalgesia, and allodynia) Antioxidant markers analysis Immunohistochemical analysis: GFAP Cleaved caspase 3 expression Quantitative real time-polymerase chain reaction RNA isolation	CV significantly mitigated the acrylamide (AA)-induced nervous system neurotoxicity. CV could provide neuroprotection to brain and sciatic tissues via its diverse influence on a number of pathways related to inflammation, apoptosis, and gene expressions.
10		[50]	Solution	40 and 70 mg/kg	21	Male Wistar rats	IP	Behavioural tests (spatial memory test in radical arm maze) Caspase-3, Bax, Bcl-2, and Bdnf gene expressions and the number of pyknotic neurons in the hippocampus were quantified	CV protected the rats against cognitive dysfunction induced by trimethyltin (TMT) chloride. CV modulated gene expression by downregulating Bax and caspase-3 and upregulating Bcl-2, attenuating the number of pyknotic neurons in the CA1 region of the hippocampus.

PO = Per os (Oral Gavage), IP = Intraperitoneal.

**Table 7:** Summary of study designs and effect of CV on anxiety, depression, and behavioural/cognitive problems.

No.	Study Type	Ref.	Formulation Type	Doses of CV	Experiment Timespan (Days)	Test Subject	Route	Analyses Techniques	Key Findings and Remarks
1	In vivo	[51]	Solution	12.5, 25, and 50 mg/ kg	<1	Male Swiss Mice	PO	Behavioural tests (elevated plus maze test, open-field test, rotarod test, pentobarbital-induced sleeping time)	Acute administration of CV at 25 and 50 mg/kg doses demonstrated a significant reduction in grooming, an indication of an anxiolytic effect in mice treated with flumazenil. However, CV at all the three tested doses demonstrated no significant effect on locomotor activity, motor coordination, sleep time, and sleep latency time in mice.
2		[52]	Solution (olive oil)	500 mg/kg	14	Wistar Rats	PO	Behavioural tests (elevated plus maze, social interaction test, Vogel test, and locomotor activity)	CV and rosmarinic acid (RA) were reported to have a moderate anxiolytic effect as compared to their mixture extract in Satureja montana. CV and RA were proposed to have neuroprotective activity mainly due to their antioxidant properties rather than any influence on the neuronal pathways. The study indirectly suggested that CV alone might not be as efficient for neuroprotection and may require a synergistic active ingredient for a prominent therapeutic effect.
3		[53]	Solution	12.5, 25, and 50 mg/ kg	<1	Male Swiss Mice	PO	Behavioural tests (forced swimming test and tail suspension test)	The anti-mobility activity of CV in the forced swimming and tail suspension tests was completely blocked off by pre-treatment of mice with SCH23390 or sulpiride as compared to yohimbine, p- chlorophenyl alanine, and prazosin. These findings suggested that the antidepressant activity of CV is somehow dependent on the dopaminergic system that was blocked by sulpiride in the mice model.
4		[54]	Solution	20, 30, and 40 mg/kg	21	Albino Wistar Rats	IP	Corticosterone evaluation Measurement of lipid peroxidation Estimation of reduced glutathione Measurements of enzymes Protein estimation	CV ameliorated the oxidative stress caused in rats in restraints. CV was reported to significantly reduce the levels of serum corticosterone (stress hormone), free radicals, and lipid peroxidation.
5		[55]	Solution	50 mg/kg	60	Sprague Dawley Rats	PO	Behavioural tests (open-field test, rod walking test, and object recognition test) Evaluation of enzymatic antioxidants (superoxide dismutase and glutathione peroxidase) Comet assay	Anorexia was observed in the PCZ control group leading to weight loss as compared to the PCZ + CV treatment groups. CV successfully countered the PCZ-induced negative neurobehavioral effects due to its antioxidant and anxiolytic properties.
6		[56]	Solution	40 mg/kg	7	Female Sprague-Dawley Rats	PO	Behavioural tests (open-field test, object recognition test, and Morris water maze test) Enzyme-linked immunosorbent assay	CV improved cognitive functioning in postmenopausal hypertensive rats. CV as a neurotrophic agent provided oestrogen-independent cognitive improvement. Apart from the conventional properties of the neuroprotective oil, CV was also found to lower blood pressure in hypertensive rats.

PO = Per os (Oral Gavage), IP = Intraperitoneal.

**Table 8:** Summary of study designs and effect of CV against LPS-challenged animal models.

No.	Study Type	Ref.	Formulation Type	Doses of CV	Experiment Timespan (Days)	Test Subject	Route	Analyses Techniques	Key Findings and Remarks
1	In vivo	[57]	Solution	25, 50, and 100 mg/kg	7	Male Wistar Rats	IP	Behavioural tests (passive avoidance test, Morris water maze test) Biochemical assessment (measurement of total thiol contents, measurement of SOD and CAT activities, measurement of MDA, measurement of NO metabolites, measurement of IL-6)	CV protected cognitive functioning against oxidative stress by lipopolysaccharides (LPS) in the rat model. Overall, the three doses of CV had the same neuroprotective efficacy; however, it was noted that highest dose of CV (100 mg/kg) elevated the thiol contents in the hippocampus more than the lower doses.
2		[58]	Solution	25, 50, and 100 mg/kg	28	Male Sprague-Dawley Rats	IP	Behavioural tests (object recognition task, Morris water maze test, open-field test) Inflammatory mediators and NF-κB measurement Total RNA isolation and RT-PCR analysis	CV was found to be a potential neuroprotective agent as it inhibited the production of inflammatory cytokines in rats with LPS-induced neurodegeneration. CV upregulated the expression of brain-derived neurotrophic factor (BDNF) mRNA and downregulated the expression of Toll-like receptor 4 (TLR4) mRNA. The highest dose of CV showed a highly positive effect on memory functioning.
3		[59]	Solution	25, 50, and 100 mg/kg	7	Rats	IP	Behavioural tests (forced swim test, open-field test, elevated plus maze) Biochemical analysis	CV was found to improve performance in behavioural tests among LPS-challenged rats. An increase in thiol content in the brain was strongly associated with the highest dose (100 mg/kg) of CV only. Malondialdehyde (MDA) levels were influenced only at higher doses (50 and 100 mg/kg), and the lowest dose of CV had no effect.
4		[60]	Solution	25 and 50 mg/kg	19	Male Wistar Rats	IP	Behavioural test (Morris water maze test) Cytokine level and thiobarbituric acid reactive substance level Total thiol concentration	CV at the lower dose (25 mg/kg) as a dietary supplement was found to be more effective in reducing tumour necrotic factor-α (TNF-α) and oxidative stress in rat brains with LPS-induced damage. CV could preserve the spatial memory functions due to its antioxidant properties.

IP = Intraperitoneal.

**Table 9:** Summary of study designs and neuroprotective potential of CV.

No.	Study Type	Ref.	Formulation Type	Doses of CV	Experiment Timespan (Days)	Test Subject	Route	Analyses Techniques	Key Findings and Remarks
1	In vitro	[61]	Nanoemulsion	25 and 50 µM	90	Peripheral blood mononuclear cell (PBMC) culture supernatants	-	Particle size, PDI, zeta potential Stability tests In vitro cytotoxicity Cytokine quantification	The biological activity of CV was found to be improved when encapsulated in a nanoemulsion. CV optimised in a nanoemulsion reduced the levels of the pro-inflammatory cytokines IL-2, IL-17, and IFN-γ at 50 µM.
2		[62]	Solution	12.5 to 800 µM	3	PC12 cell-based neuronal model	-	MTT assay Neurite outgrowth Inhibition of NGF-signalling pathway NGF expression Western blot Immunofluorescence staining of NF-200	CV was found to stimulate the neurotrophic pathways that create axonal and synaptic plasticity without depending on nerve growth factor (NGF). Cell viability studies illustrated that 12.5 to 200 µM of CV were safe, and any concentration greater than this was cytotoxic (highest = 800 µM). CV may preserve or regenerate axons, a potential gamechanger in neurodegenerative diseases.
3	In vivo	[63]	Solution	10 µg (i.c.v) and 5, 25, and 50 mg/kg (IP)	<1	Male ICR mice	ICV and IP	Neurological deficit scoring evaluation Detection of infarction volume Western blot analysis	Potential therapeutic neuroprotective strength of CV depends on the method of administration. The neuroprotective outcome was found to be greater when administered directly into the cerebroventricular cavity than when administered intraperitoneally. CV demonstrated anti-apoptotic activity and was found to have an extended therapeutic window when it maintained its protective effect even when it was administered six hours after reperfusion.
4		[64]	Solution (peanut oil)	12.5 mg /kg for 7 days and 150 or 450 mg/kg for acute single doses	7	Male Wistar rats	PO	Measurement of monoamine neurotransmitter levels Behavioural test (forced swimming test)	CV has the potential to modulate brain regions depending on the administered concentrations and timespan. CV was found to increase the dopamine levels in the prefrontal cortex and hippocampus at low doses (12.5 mg/kg). On the contrary, higher acute doses (450 mg/kg) of CV were associated with a lowering of dopamine levels in the hippocampus.
5		[65]	Solution	25, 50, 75, and 100 mg/kg	7	Male C57BL/6 mice	IP	Behavioural testing Measurement of brain water content Measurement of Evans Blue Immunohistological staining and imaging Relative quantitative real-time PCR analysis Western blot analysis	CV at 100 mg/kg reduced cerebral oedema when administered 1 h after induing intracerebral haemorrhage (ICH) in mice and had no effect when administered after 3 h of ICH. CV regulated AQP4 expression, a pathway responsible for cerebral oedema after injury. The authors emphasised that the route of administration and time of CV treatment is important in determining the neuroprotective effects of EOs in brain injuries.
6		[66]	Solution	3 mM	3	Male Sprague-Dawley rats	-	Immunohistochemistry Western blot analysis Morphometric indices	CV inhibited the TRPM7 pathway and was observed to reduce axonal degradation in peripheral nerves. Myelin fragmentation was found to be significantly delayed by CV treatment. Contrary to some of the past literature, the authors hypothesised that CV might not be an inhibitor of acetylcholine receptors (AchRs) in Schwann cells.
7		[67]	Solution	25, 50, and 100 mg/kg	40	Male Wistar rats	PO	Behavioural test (Morris water maze test) Biochemical analysis (measurement of MDA, SOD, and catalase; blood lead concentration) Histological assessment of the hippocampus	The neurodegenerative effects of lead were found to be prevented by CV treatment, as evidenced from the improved memory and learning in treatment groups. Lipid peroxidation was reduced by CV, and higher doses (50 and 100 mg/kg) were found to be more effective.
8		[68]	Solution	15–30 mg/kg	28	Rats	IP	Behavioural tests (Morris water maze and passive avoidance tests) Biochemical analysis (Malondialdehyde test and thiol groups analysis)	CV decreased the MDA levels and increased the thiol content in aged rats and was reported to be associated with improved memory and learning. The authors attributed these effects of CV to its inherent antioxidant properties.
9	In vitro and in vivo	[69]	Solution	>200 mM in cell cultures and 30 or 50 mg/kg in vivo	7	Timed pregnant CD1 mice and HEK293 cell cultures	IP	Electrophysiology recording Infarct volume evaluation Whole-brain imaging and histology Immunofluorescence imaging Neurobehavioral evaluation (negative geotaxis, cliff avoidance, grip test) and Western blotting	CV could potentially protect against neonatal hypoxia in the case of a brain stroke. CV inhibited the pro-apoptotic signals, reduced brain infarct volumes, and enhanced pro-survival signals in neonate mice brains.

PO = Per os (Oral Gavage), IP = Intraperitoneal, ICV = Intracerebroventricular.
